# Energy and Exergy Analysis of an Absorption and Mechanical System for a Dehumidification Unit in a Gelatin Factory †

**DOI:** 10.3390/e23040415

**Published:** 2021-03-31

**Authors:** Lucas Sandoli Lima, Carlos Eduardo Keutenedjian Mady

**Affiliations:** 1 School of Mechanical Engineering, University of Campinas, Mendeleyev St., 200—Cidade Universitária, Campinas 13083-970, Brazil; lucassandoli@hotmail.com; 2 Department of Mechanical Engineering, Centro Universitário da FEI, São Bernardo do Campo 09850-901, Brazil

**Keywords:** absorption unit, air dehumidification, desiccant system, exergy analysis

## Abstract

In this paper, an energy and exergy analysis is applied to the air dehumidification unit of a liquid desiccant system in an industrial gelatin conveyor dryer. The working fluid is a binary solution of lithium chloride (LiCl) in water. Dry air is used in order to decrease the amount of liquid in the gelatin. Therefore, the environmental air must have its absolute humidity reduced from about 12 g/kg to the project target, which is 5 g/kg. The process is a cycle using an absorption desiccant unit (LiCl in water), where the weak solution absorbs water vapor from the air. In the regenerator, condensation of the solution (desorption) from the moist air occurs. As a result, the steam consumption of the desorber and electrical power used for the vapor compression chiller (with ammonia, NH${}_{3}$, as working fluid) are the primary sources of cost for the factory. To improve the plant’s energy and exergy behaviors, the process is evaluated using a mathematical model of the system processes. In addition, we evaluate the substitution of the vapor compression chiller by an absorption unit (lithium bromide (LiBr) in water). The performance indicators of the compression vapor systems showed the best results. Even when using the condenser’s energy to pre-heat the solution, the installed system proved to be more effective.

## 1. Introduction

Gelatin is essentially a protein based on the partial hydrolysis of an animal collagen structure. The three large-scale raw materials used for the industrial production of gelatin are bovine hide split, bovine ossein, and pigskin. Due to the abundant supply of cattle in Brazil, the hide split is the most used feedstock, which passes through several steps until it reaches the final product, gelatin. The raw material used influences several processes involved in the production. For hide split gelatin, the involved steps are: conditioning, raw material treatment, extraction, filtration, clarification, deionization, concentration, drying, and packing [1]. The process of gelatin production begins with conditioning and raw material treatment, where the leather undergoes an acid or alkaline treatment. This treatment aims to ensure the quality of the final product and to prepare the raw material. Extraction, the next step, involves the hydrolysis of the collagen. High-temperature water dissolves the hide and transforms it into gelatin. Solid particles are removed and the color is improved by the filtration and clarification steps. Deionization is essential to remove salts from the final product, as well as to improve the odor. In the concentration and drying process, the solid content of gelatin has to increase to about 90%. The final step is packing, where the product is prepared for the customer. High amounts of electricity and steam are required, which vary as a function of the technology employed and feedstock, therefore relating to the country of manufacture. These processes are, however, highly energy-intensive. As a result, to produce 1 kg of gelatin using an alkaline treatment, the steam consumption may reach values as high as 20–25 kg, requiring 3–5 kWh (10.8 to 18 MJ) of electricity [1].

The steps with the highest energy consumption are the concentration of the solution [2] and drying [3] steps. The first takes place using membrane technology [4] or multi-effect water evaporation [2], while the second takes place under a dehumidification system (in order to improve the evaporation in a conveyor dryer, it is necessary to use dry air).

One feasible route to decrease the quantity of water in the air is to decrease its temperature. Research conducted by the U.S. Department of Energy considered that 15% of the total energy expenditure in the world is due to air conditioning and refrigeration [5]. Another matter is related to the refrigeration industry, regarding the use of cooling fluids [5]. A liquid desiccant absorption system is one option for a vapor compression cooling system [6,7,8]. A Previous investigation [9] has shown that it is possible to achieve a reduction in overall energy consumption, although it has high initial system costs [9]. For industrial applications (e.g., in food industries), while a conventional vapor compression system simultaneously refrigerates and dehumidifies the air, a desiccant system only dehumidifies it, which may decrease the energy consumption of the plant [10]. There are three main varieties used: liquid, solid, and advanced desiccants (i.e., polymeric, composite, or bio-desiccants). Air with low absolute humidity can increase the production of a dryer. Other options for this technology (liquid desiccant) are the usual vapor compression systems and desiccant wheels [10].

The benefits of using liquid desiccant, in comparison to other fluids are: When released to the environment, these solutions have low GWP (Global Warming Potential) and ODP (Ozone Depletion Potential); the associated systems have lower energy consumption than evaporative cooling systems; and they incur a lower initial cost, compared to desiccant wheels. There are, however, some disadvantages, such as the corrosive capability of desiccant fluid when the solute is LiCl or LiBr [10]. Some additional studies comparing glycols and salt solutions (e.g., LiCl, LiBr, and other pairs) have been carried out [11], demonstrating the advantages of some salt solutions; for example, they do not vaporize in environmental conditions. In addition, these solutions have good performance, especially with the use of LiCl and LiBr, showing little difference [11]. These comparisons have been carried out in the literature through several studies [12,13], even using solar energy [14].

Wang et al. [15] have presented an exergy analysis of such systems, involving an evaluation of the exergy losses for different dehumidification temperatures and regeneration temperatures. The exergy analysis of these systems has been a focus in the literature in recent years, as in [16,17,18].

In this article, the real industrial complex of a gelatin plant of the Gelita Group, located in Mococa in São Paulo State, Brazil, is evaluated. The process chosen was the dehumidification/regeneration system. The system performance was evaluated from the point of view of the First and Second Laws of Thermodynamics. In addition, we analyzed the replacement of the vapor compression refrigeration system by an absorption system, as steam is already produced within the plant. Additionally, a distinguishing feature of this article is presenting a complete analysis of desiccant systems.

## 2. Methods

### 2.1. Process Representation

A liquid desiccant dehumidifier/regenerator is a system using an absorption solution which has a packing bed for dehumidification, where the phenomenological model is out of the scope of this article and has been studied previously in the literature, such as [13]. The main goal was to decrease the absolute humidity of the air. There is a second packing bed for regeneration, where the water in the dehumidifier is eliminated. The liquid desiccant plays an essential factor in the performance of the drying air system. The desiccant solution has two alternatives, in order to improve the mass transfer of water from the air: The first one is to decrease the temperature of the brine before entering the absorber (dehumidifier), while the second involves raising the concentration of the brine (this latter approach has a limitation regarding the crystallization temperature).

**Figure 1:**
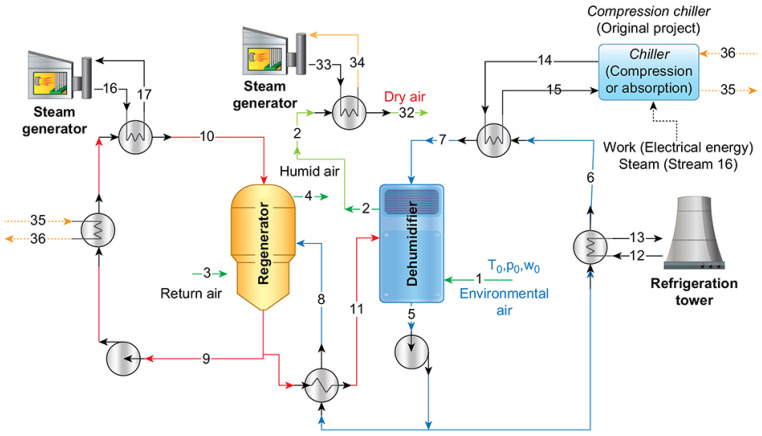
Process diagram of the liquid desiccant system (using an LiCl–H${}_{2}$O mixture) to decrease the humidity of the air before air drying.

Figure 1 demonstrates the processes used to obtain air with low absolute humidity. The dehumidifier and regenerator are presented in the center. Streams 16 and 33 are the saturated vapor generated in the boilers and, to refrigerate the solution, the system originally used a vapor compression chiller (already used in the installation) with a possible substitution by an absorption refrigeration system, as stream 17 has high exergy content. The environmental conditions change during the year, such that there is a necessity for a fixed thermodynamic state of the air entering the dryer (stream 32). Therefore, the desiccant system was designed to automatically change the parameters to ensure the setpoint, which is the absolute humidity of the dry air (stream 2). The liquid desiccant applied in the system is a solution of lithium chloride in water (LiCl–H${}_{2}$O); the strong solution has a concentration of 45% (streams 9 to 11) and the weak solution 42% (streams 5 to 7). These concentrations are higher than usual systems [6]; nevertheless, they resulted in the best parameters for this specific system. One other point is the possibility of using the energy content in the chiller’s condenser to preheat the LiCl-H${}_{2}$O solution between streams 9 and 10. These residual exergies are shown as streams 35 and 36 and would be just lost (or destroyed) in the environment.

Regarding Figure 1, stream 1 is the air entering the system (considered as the reference state for the application of the exergy analysis), while stream 2 is the output (or product) of the system, later heated to stream 32. There is still a necessity to increase the air temperature. Therefore, the air that goes to the dryer—stream 32—must be heated, requiring energy expenditure in this step.

The air entering in stream 3 is the return air leaving the dryer system (not shown in the Figure), which is used for regeneration. The air leaves in stream 4 and receives the water condensed in the dehumidifier. The usual dehumidification process (i.e., with a liquid desiccant) requires the fluid temperature to be decreased in the absorber (e.g., cooling tower, refrigeration chiller) and a source with high temperature in the regenerator. The primary sources of operational costs are related to these operations (steam production and decreased temperature from streams 6 to 7).

**Figure 2:**
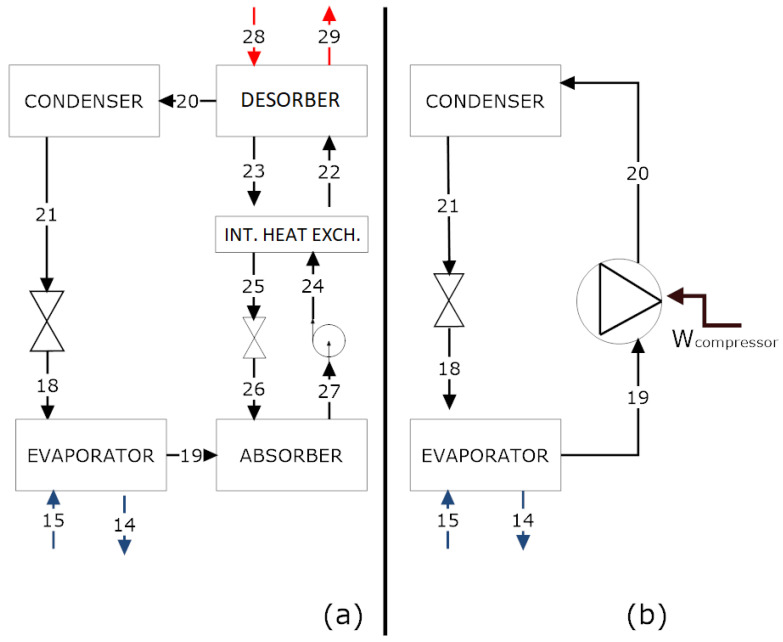
(**a**) Absorption chiller with LiBr–H${}_{2}$0 and (**b**) vapor compression chiller operating with NH${}_{3}$.

In the dehumidifier of the considered gelatin factory, the current chiller is an ammonia vapor compression cycle (Figure 2b). There exists the possibility to utilize the lithium bromide in a water absorption chiller (Figure 2a), as the factory already produces its own steam (or hot liquid water, as in stream 17). Independent of the technology, the evaporators in both arrangements will exchange the same amount of heat (therefore, carrying the same thermal load), and the streams, when cooled down, will be the same. The main difference between the two chillers is the energy source, as one uses electrical energy, while the other uses thermal energy. Generally, an absorption chiller is more feasible when a residual energy source is available.

The vapor compression system uses ammonia as a working fluid and employs a reciprocating compressor. When the vapor leaves the evaporator (stream 19), the compressor forces the fluid to high pressure (stream 20). Afterward, the refrigerant is cooled down in the condenser (stream 21) and, finally, the throttling valve carries out expansion to enable the refrigeration step again (stream 18).

The absorption chiller avoids compression in the refrigeration process. To replace this process, we considered a working pair. In this study, the working pair is an LiBr–H${}_{2}$0 solution (which differs from that of the desiccant system). Water is the refrigeration fluid. A schematic representation of the chiller is given in Figure 2a. The only need for electrical en ergy is in the pump, which has low energy expenditure (i.e., negligible specific volume, compared with the vapor compression system).

### 2.2. Thermodynamic Model

To properly apply the exergy analysis, it is necessary to evaluate the mass conservation and energy balance of each component of the cycle. The state of each stream was calculated with data collected from the gelatin factory; some balances respect the principles described in this section. Mass conservation can be expressed by Equations (1) and (2). The latter equation is for a binary mixture of a salt (e.g., LiBr or LiCl) in water, with a given mass concentration $x_{i}$.

(1) $$\sum_{in}{\dot{m}}_{in}-\sum_{out}{\dot{m}}_{out}=0,$$

(2) $$\sum_{in}x_{in}{\dot{m}}_{in}-\sum_{out}x_{out}{\dot{m}}_{out}=0.$$

The energy conservation of each piece of equipment (for steady state operations) in the system is expressed by Equation (3):

(3) $${\dot{Q}}_{CV}-{\dot{W}}_{CV}=\sum_{out}\left({\dot{m}}_{out}h_{out}\right)-\sum_{in}\left({\dot{m}}_{in}h_{in}\right),$$

where the enthalpy, heat transfer rate, and power are represented by, *h*, ${\dot{Q}}_{CV}$, and ${\dot{W}}_{CV}$, respectively.

The exergy analysis is carried out using Equation (4), where ${\dot{B}}_{d}$ stands for the irreversibilities or destroyed exergy and *b* stands for the specific exergy of a stream, determined by the sum of physical and chemical exergy: $b=b_{physical}+b_{chemical}$.

(4) $${\dot{B}}_{d}=\sum_{in}{\dot{B}}_{in}-\sum_{out}{\dot{B}}_{out}+\sum_{k}{\dot{Q}}_{k}\left(1-\frac{T_{0}}{T_{k}}\right)-{\dot{W}}_{CV}.$$

The exergy of stream *i* is given by Equation (5), according to [19,20]:

(5) $${\dot{B}}_{i}={\dot{B}}_{physical,i}+{\dot{B}}_{chemical,i}.$$

The physical exergy, for a known reference environment, $T_{0}$, and $P_{0}$, is given by Equation (6). The chemical exergy of a mixture was evaluated according to Szargut et al. [19].

(6) $${\dot{B}}_{physical}=\dot{m}\left[h-h_{0}^{*}-T_{0}\left(s-s_{0}^{*}\right)\right],$$

(7) $${\dot{B}}_{chemical}=\dot{n}\sum_{i=1}^{k}x_{i}\left[{\bar{\mu }}_{i}^{*}\left(T_{0},p_{0},x_{i}\right)-{\bar{\mu }}_{i}^{*}\left(T_{0},p_{0},x_{i,0}\right)\right].$$

In order to evaluate the chemical exergy of a mixture, several articles have discussed the methods and reference conditions [17,18,21]. The article written by Oliveira-Junior and Le Goff [18] assessed the equilibrium to the dead state, where: (i) for the binary mixture in cycles, the reference was determined as the solute with a concentration of 20%; and (ii) for the pure solvent, the substance at the dead state [20]. Other articles have discussed the necessity of the chemical exergy in evaluating the thermodynamic behavior of the cycle. These values have been determined in [21], where the error associated with considering (or not) the chemical exergy of the mixture was only significant in the absorber and generator [22,23]. One article has even compared all these methods, as proposed by Palacios et al. [17], in which it was shown that the method of Oliveira-Junior and Le Goff [18], indeed, may be used to analyze the exergy behavior of a binary mixture. The authors also carried out this approach in [24].

From the mass, species, and energy balances, it is possible to assess the energy behavior of the desiccant cycle. The so-called phenomenological model [25,26] operates with two refrigeration cycles: absorption and vapor compression. Equations (8) and (9) indicate the performance coefficient, based on the First Law of Thermodynamics; nevertheless, these two indices provide a clue about how the cycle is operating, compared to one of the same nature, which has also been discussed in [27]. In contrast, the comparison of both cycles requires the application of exergy analysis. The energy intake of the product is the heat transfer removed from the solution (${\dot{Q}}_{evaporator}$) in Figure 1 in streams 6 to 7. The cycles are indicated in Figure 2a,b. The energy input is the compression power, ${\dot{W}}_{compressor}$, while that for the absorption cycle is ${\dot{Q}}_{desorber}$.

For the cooling cycle, an energy parameter for efficiency is the coefficient of performance (COP), which is the ratio of heat load in the evaporator to the energy input of the system. For the absorption system, the COP is evaluated by Equation (8) (neglecting the work of the pump); whereas, for the compression system, the COP is evaluated by Equation (9).

(8) $$COP_{compression}=\frac{{\dot{Q}}_{evaporator}}{{\dot{W}}_{compressor}},$$

(9) $$COP_{absorption}=\frac{{\dot{Q}}_{evaporator}}{\left({\dot{Q}}_{generator}+{\dot{W}}_{pump}\right)}.$$

The exergy efficiency can be used to compare the same solution [28]; nevertheless, it can also be used under different operational conditions and has been considered a proper tool to evaluate two different solutions [29]. The exergy efficiency may be evaluated according to Equations (10) and (11). In both scenarios, the desired effect is considered as the exergy of the stream 6 to 7 (${\dot{B}}_{Q_{evaporator}}$), where the exergy input is considered as ${\dot{W}}_{compressor}$ and ${\dot{B}}_{Q_{desorber}}$, and ${\dot{W}}_{pump}$ may be considered negligible when the absorption chiller is using the LiBr–H${}_{2}$O solution [30].

(10) $$\eta_{ex,compression}=\frac{{\dot{B}}_{Q_{evaporator}}}{{\dot{W}}_{compressor}},$$

(11) $$\eta_{ex,absorption}=\frac{{\dot{B}}_{Q_{evaporator}}}{\left({\dot{B}}_{Q_{desorber}}+{\dot{W}}_{pump}\right)}.$$

Another usual parameter used for this type of device is the amount of water removed from the air (i.e., the difference between streams 1 and 2) divided by the energy input $\dot{E}$, therefore g${}_{water}$/kJ${}_{energy}$, according to Equation (12). A modification of this index is introduced based on exergy analysis, determined by Equation (13). Therefore, the parameter SMER is defined as a specific moisture extraction rate in an energy (SMER${}_{en}$) and an exergy basis (SMER${}_{ex}$).

(12) $$SMER=\frac{{\dot{m}}_{water,removed}}{{\dot{E}}_{input}}$$

(13) $$SMER_{ex}=\frac{{\dot{m}}_{water,removed}}{{\dot{B}}_{input}}$$

Equations (12) and (13) raise light into a proper overall definition of the liquid desiccant system’s exergy efficiency. It is essential to highlight that there are two exergy inputs: (i) exergy transfer by the steam generator (herein considered only as of the $\Delta {\dot{B}}_{steam}$) and (ii) the exergy transfer by the refrigeration cycle to achieve sub-environmental temperatures (${\dot{B}}_{Q_{evaporator}}$). Therefore, Equation (14) indicates the calculus of the system’s exergy efficiency.

(14) $$\eta_{ex}=\frac{\Delta {\dot{B}}_{air}}{{\dot{B}}_{input}}$$

## 3. Results and Discussions

### Comparison of Both Solutions

For each stream, mass, species, energy, and exergy analyses were applied, in order to obtain the thermodynamic states for the desiccant absorption chiller with the LiCl–H${}_{2}$O solution and, for the refrigeration, the chiller using ammonia and the binary pair LiBr–H${}_{2}$O. The software used was Equation Engineering Solver (EES) [31], due to its thermodynamic properties.

Table 1 indicates the thermodynamic states of the desiccant absorption system and operational data of the plant. This was considered as the reference scenario, regarding Figure 1 and Figure 2a,b. Stream 1 is the entering air removed from the environment. The reference conditions are considered with an established temperature, pressure, and humidity ratio (0.016 $kg_{v}/kg_{a}$) for this entire article. Index 0 expresses this thermodynamic state. This fact supports the exergy of the entering air to have negative marks.

In the last column of Table 1, the fluid representing the stream is indicated. The specific enthalpy and entropy were obtained directly from Thermodynamic properties and, for the specific exergy of the stream, we considered that the reference condition was 20% (in mass) for LiCl/LiBr–H${}_{2}$O solutions [17,18]. It is important to highlight the necessity of the use of a refrigeration tower and, in some conditions, a refrigeration cycle; such as the situations indicated in Figure 2a,b. In the original configuration of the plant, the vapor compression chiller was used [32]; herein, we tested the absorption chiller based on the LiBr–H${}_{2}$O pair [30].

Concerning the Second Law of Thermodynamics, all exergy balances were calculated using Equation (4). With the exergy defined for all states, irreversibilities were evaluated for each component of the system. In Table 2, the destroyed exergy of each piece of equipment is given (as well as its relative participation in the total amount of destroyed exergy). It is essential to pronounce here that we did not take into account the irreversibilities of the combustion and the refrigeration systems, which will be further analyzed. When we assessed the irreversibilities of the desiccant system solely, we noticed that the greatest destructions of exergy were by the steam generation heat exchanger and the absorber. It is required to accentuate that the steam heat exchanger represented the largest exergy destroyed (without taking into consideration the combustion process), the main reason for this being the temperature difference between the solution and vapor streams. Concerning the absorber, the leading cause of the exergy destruction rate was the process of mixing between the air and the solution, which occurred inside the chamber. In addition, there were losses in the process related to heat and mass transfer. The overall exergy destruction of the system was 235.50 kW. The exergy input is $\Delta {\dot{B}}_{steam}={\dot{B}}_{16}-{\dot{B}}_{17}=253.81$ kW and the useful exergy is $\Delta {\dot{B}}_{air}={\dot{B}}_{32}-{\dot{B}}_{1}=$ 25,405 kW and the exergy efficiency was 10% (ratio of the exergy of the air to the provided exergy). Note that taking into consideration only the liquid desiccant system, the increase in the exergy from streams 6 to 7 is almost negligible.

In Table 3, the thermodynamic states of the steam compression chiller are given. Some parameters were obtained in the factory, such as the steam temperature leaving the compressor. We also gathered the saturation pressures of the condenser and evaporator [32]. It is possible to notice that the temperature of the ammonia in the evaporator was around 10 ${}^{{}^{\circ}}$C, in order to decrease the temperature of the LiCl–H${}_{2}$O solution from 29 ${}^{{}^{\circ}}$C to 23 ${}^{{}^{\circ}}$C, considering the design conditions (25 ${}^{{}^{\circ}}$C). Depending on the environmental circumstances, there was no requirement to utilize a refrigeration cycle.

From the thermodynamic states, it was possible to evaluate the exergy destroyed in each piece of equipment (Table 4).

For this study, we intended to compare the use of an absorption chiller, as opposed to a vapor compression system, in terms of energy and exergy usage. The absorption chiller can be used as an alternative to the vapor compression system as, in most chemical plants, steam is produced within the factory. Therefore, the use of an absorption chiller was a suitable option. The LiBr–H${}_{2}$O solution obtained the same effect between the input and output states of the vapor compression process. Generally, the energy source used for these systems is steam or water with high temperature; in the present analysis, we considered the prime. Table 5 indicates the thermodynamic states of the cycle.

Steam was produced inside the considered industrial complex, which increased the appeal of the use of an absorption chiller. As usually displayed in chiller catalogs, the cold water was fixed in a temperature range of 7–12 ${}^{{}^{\circ}}$C (streams 14 and 15), based on the literature. From the energy balance in the evaporator, the result was a temperature of 12 ${}^{{}^{\circ}}$C of the water (refrigerant) before entering the absorber (streams 18 and 19). Table 5 indicates the thermodynamic states of each stream in the cycle. The concentrations adopted for the solution were 56.7% and 62.4%, according to the literature [30]. Our intent was to keep the pressure of the fluid and flow rate in values close to that of the few manufacturers found in the literature.

Evaluating the system’s exergy behavior, Table 6 shows that the greatest irreversibilities were in the desorber, followed by the absorber, with exergy destruction due to the mixing/separation and heat transfer rates as the primary sources. Expanding the dehumidifying system frontiers to include the absorption chiller, the global efficiency was found to be 7.16%. The performance coefficient COP of the refrigeration cycle was 0.7463, while the SMERex was 2.295 kg/kWh. The irreversibility results are shown in Table 6. The total exergy losses were found to be 331.76 kW (dehumidifier and absorption chiller).

As already demonstrated through the states defined for the inlet air temperature at 25 ${}^{{}^{\circ}}$C and absolute humidity of 0.013 $kg_{v}/kg_{a}$, in this case, the system using a vapor compression chiller showed irreversibility, exergy efficiency, and SMERex index, respectively, of 256.96 kW, 9.05%, and 2.796 kg/kWh. In contrast, those of the absorption chiller were 331.76 kW, 7.16%, and 2.295 kg/kWh. Therefore, the desiccant liquid absorption dehumidifier with the vapor compression chiller proved to be more viable and the best alternative between the two studied. As the system is used throughout all seasons of the year, in different periods of the day, and with the most varied parameters, another evaluation could be done to check whether, under other operating points with different temperatures and air humidities, the absorption chiller would be more efficient. The figures below show the exergy efficiencies at 30 °C, 25 °C, and 20 °C, with different relative humidities for the environmental air.

**Figure 3:**
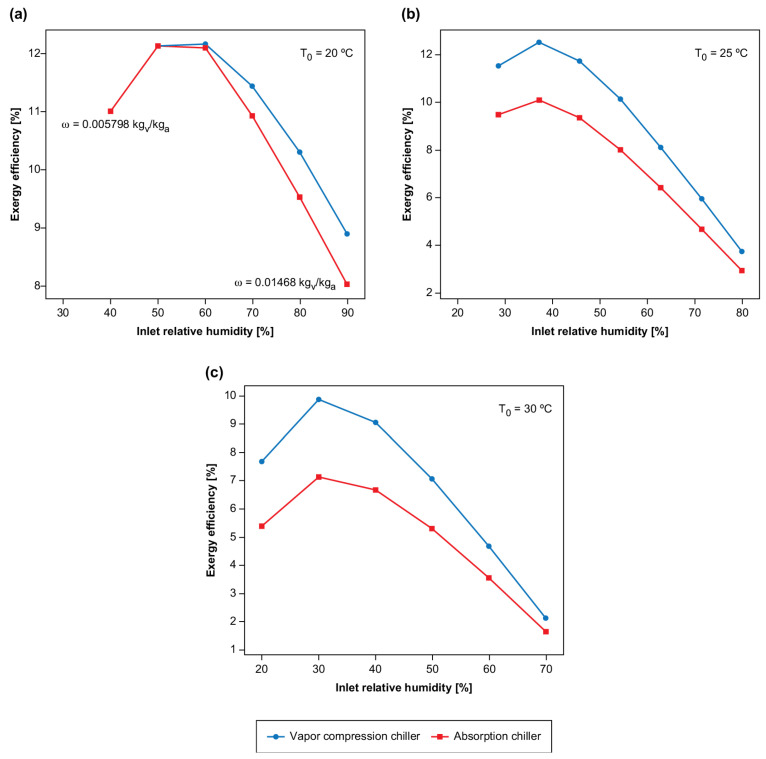
Exergy efficiency of both dehumidification solutions, using absorption and compression chillers as a function of relative humidity and air temperature (20, 25, and 30 ${}^{{}^{\circ}}$C named Figures (**a**), (**b**), (**c**); respectively).

Figure 3 shows a comparison of the exergy efficiency as a function of the relative humidity for three inlet temperatures (20, 25, and 30 ${}^{{}^{\circ}}$C). Therefore, from the comparison, Figure 3a–c show that an increase in the inlet temperature of the air (environmental temperature) caused the chillers to operate using higher power (electric) or enthalpy (steam). It is interesting to note that, at 20 ${}^{{}^{\circ}}$C, both systems had the same efficiency (ϕ<50%), as there was no necessity for the chiller (i.e., they were turned off), and only the refrigeration tower was operated.

As shown in Figure 3a–c, for all temperature ranges (30, 25, and 20 ${}^{{}^{\circ}}$C), the system with the vapor compression chiller presented higher exergy efficiency for all values of relative humidity. Therefore, it appears that, for all studied temperature and humidity ranges, the steam absorption chiller, as an energy source, does not offer thermodynamic viability.

It is, however, essential to note that the two systems showed the same efficiencies for the lowest temperature and humidity. This is because, at low humidity and temperatures (temperature and relative humidity below 20 °C and 50%), it was not necessary to use the chiller to cool the inlet solution in the dehumidifier: it was possible to reach humidities below those necessary for the system using only the cooling tower.

Therefore, a possible way to increase the system’s efficiency is to improve the cooling tower’s performance as, the better the thermal exchange, the lower the solution’s mass flow rate needs to be cooled by the chiller. Another point that can be observed in the charts is that the absorption systems, through the liquid desiccant, increase in efficiency as the relative humidity decreases until reaching a maximum point and, then, their efficiency decreases again; that is, this system is more suitable for use in environments that are in “intermediate conditions”. The explanation for this issue is that the thermal load to decrease the air temperature is almost the same (since the air temperature difference would be the same). Although the amount of water removed would be lower for relative humidity reduced, it justifies both systems a decrease in the exergy efficiency. The exergy provided is the same, and the increase in the exergy of the air is lower.

**Figure 4:**
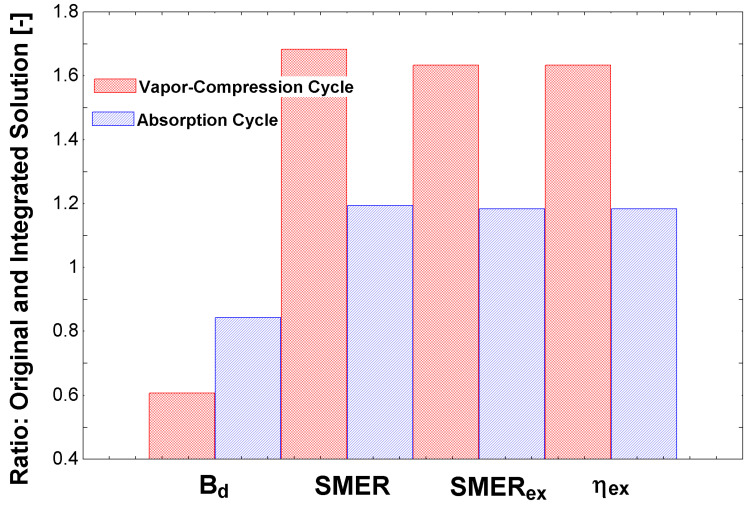
Comparison of indices using the integrated solutions for both types of cycles: compression and absorption.

Figure 4 shows a comparison of the compression cycle and absorption cycle in the whole system (complete dehumidification system), with and without an usual integration in the air conditioning area of the streams 35 and 36 to preheat the solution before the steam generator. It is essential to highlight that this is not a comprehensive integration, only a solution to reuse the condenser’s exergy content. The results are show in comparison with the project scenario shown in Table 1. Therefore, using the condenser’s energy to preheat the solution before the boiling entrance (i.e., from stream 9 to 10) increased all performance indicators. It is essential to indicate the similarity of behavior of $SMER_{ex}$ and $eta_{e}x$, as both used exact quantities to propose the associated performance indices. This similar trend was expected. The traditional SMER index had identical behavior, although at another magnitude. This result shows the importance of comparing these different parameters, in order to conclude the best solution. In these two scenarios, with specific conditions, the original compression cycle would be more appropriate.

It is important to discuss that, in this figure, the usage of the condenser’s energy (which would be transferred to the environment as an exergy loss) is now used as an input to the process. Another critical point is the higher exergy performance of the compression cycle using ammonia. Future research may indicate which energy input would be better to invest in, e.g., photovoltaic for the compressors or solar thermal energy for the absorption cycle, or another process that requires steam. From the comparison of both refrigeration systems, it is possible to conclude that there was a considerable discrepancy between the chillers: The vapor compression system had an advantage concerning the performance coefficients. Therefore, there was an unfavorable point hidden in these production indicators. The absorption chiller, in this specific case, used steam produced in the plant’s boiler. Therefore, it was considered a high-quality energy input, which may be suppressed by other energy sources of lesser quality, such as residual heat, natural gas, or renewable energy. Of course, the quality of the energy input to the compression chiller was high; nevertheless, it may not use other production types, other than photovoltaic cells. A comparison of both may be carried out in a future study.

## 4. Concluding Remarks

The system studied was a dehumidifier utilizing the principle of moisture absorption by use of a desiccant liquid (a mixture of LiCl and H${}_{2}$O). An alternative was evaluated for this system: The replacement of the vapor compression chiller (with NH${}_{3}$), in order to reduce the temperature of the solution that absorbs water in the conditioner. The plant has a source of so-called “thermal energy” through steam (i.e., enthalpy variation of the steam). An alternative would be to replace the electric power used in the original system’s chiller using an absorption chiller with LiBr in H${}_{2}$O.

From the analysis proposed in this article, the refrigeration compression system with NH${}_{3}$ demonstrated better performance indicators, compared with the absorption chiller for the technologies studied in the present article (without taking into account renewable energy production instead of using boilers), as the destroyed exergy in the absorption chiller was considerably higher.

When a sensibility analysis was carried out, an inserting behavior was observed. There was a point of relative humidity and environmental temperature at which the conditioning systems were turned off, where only the refrigeration tower was used to decrease the solution temperature. This indicates that the location at which the plant is installed influences the system’s exergy behavior. In addition, an improvement in the behavior of the tower could decrease refrigeration costs drastically.

Eventually, with both systems turned on, there is a point of maximum exergy efficiency with moderate relative humidities. Additionally, the integration of the vapor compression system’s condenser could improve the performance of the overall system. This effect was more pronounced for the project system’s operational conditions than that of the absorption chiller.

## Figures and Tables

**Table 1:** Desiccant absorption system of LiCl–H${}_{2}$O thermodynamic states. Pressure, temperature, specific enthalpy, specific entropy, mass flow rate and absolute humidity of the air, concentration of binary mixture, and exergy rate.

State	*P* (kPa)	*T* (${}^{{}^{\circ}}$C)	*h* (kJ/kg)	*s* (kJ/kg.K)	$\dot{m}$ (kg/s)	ω (kg/kg)	x (%)	$\dot{B}$ (kW)	Fluid
0	101.3	25				0.016	20		
1	101.3	25	58.28	5.815	32.21	0.013		−2.705	Air
2	101.3	25.15	37.47	5.742	31.94	0.0048		20.14	Air
3	101.3	65	117	6.004	17.34	0.01967		49.11	Air
4	101.3	72.31	163.7	6.156	17.61	0.0345		109.5	Air
5	101.3	38.08	169.7	0.333	19.36		42	16,065	LiCl-H${}_{2}$O
6	101.3	29.03	145.6	0.2547	19.36		42	16,052	LiCl-H${}_{2}$O
7	101.3	23	129.5	0.2007	19.36		42	16,051	LiCl-H${}_{2}$O
8	101.3	60.73	228.9	0.5168	3.766		42	3143	LiCl-H${}_{2}$O
9	101.3	69.62	274	0.5707	17		45	15,559	LiCl-H${}_{2}$O
10	101.3	92	330	0.7288	17		45	15,708	LiCl-H${}_{2}$O
11	101.3	44.39	210.3	0.3774	3.5		45	3182	LiCl-H${}_{2}$O
12	101.3	23	96.48	0.3388	30			1501	H${}_{2}$O
13	101.3	26.7	112	0.3908	30			1501	H${}_{2}$O
14	201.3	15.02	63.19	0.2245	15			762.2	H${}_{2}$O
15	201.3	20	84.02	0.2962	15			754.2	H${}_{2}$O
16	300	133.6	2725	6.992	0.4394			305.5	H${}_{2}$O
17	300	133.6	561.6	1.672	0.4394			51.69	H${}_{2}$O
32	101.3	32	44.42	5.765	31.94	0.0048		22.87	Air (product)
33	300	133.6	2725	6.992	0.1027			71.42	H${}_{2}$O
34	300	133.6	561.6	1.672	0.1027			12.08	H${}_{2}$O
35	300	133.6	2725	6.992	0.1027			71.42	H${}_{2}$O
36	300	133.6	561.6	1.672	0.1027			12.08	H${}_{2}$O

**Table 2:** Destroyed exergy of the desiccant liquid system, not accounting for the external irreversibilities to the process.

Equipment	${\dot{B}}_{d}$ (kW)	%
Regenerator	29.0	12.3
Dehumidifier	18.8	8.0
Chiller Heat Exchanger	9.1	3.9
Intermediate Heat Exchanger	4.7	2.0
Tower Heat Exchanger	13.2	5.6
Steam Generation Heat Exchanger	104.1	44.2
Heater	56.6	24.0
Total	235.5	100.0

**Table 3:** Thermodynamic states of the vapor compression chiller, with data obtained in the plant and based on [32].

Stream	P (kPa)	T (${}^{{}^{\circ}}$C)	*h* (kJ/kg)	*s* (kJ/kg.K)	$\dot{m}$ (kg/s)	$\dot{B}$ (kW)
18	615.7	10.02	341.8	1.503	0.2764	5572
19	615.7	10.02	1472	5.494	0.2764	5555
20	1167	62.35	1581	5.56	0.2764	5580
21	1167	30	341.8	1.488	0.2764	5573

**Table 4:** Overall destroyed exergy rate (total) and destroyed exergy rate for each component in the vapor compression system.

Equipment	${\dot{B}}_{d}$ (kW)	Relative Values%
Compressor	8.4	39.1
Valve	1.19	5.5
Evaporator	8.46	39.4
Condenser	3.42	15.9
Total	21.47	100

**Table 5:** Thermodynamic states of the absorption chiller, based on [30].

Stream	P (kPa)	T (${}^{{}^{\circ}}$C)	*h* (kJ/kg)	*s* (kJ/kgK)	$\dot{m}$ (kg/s)	x (%)	$\dot{B}$ (kW)
18	1.405	12.02	146.6	0.5179	0.1315		6.147
19	1.405	12.02	2523	8.85	0.1315		−8.075
20	5.627	80	2650	8.609	0.1315		18.06
21	5.627	35	146.6	0.505	0.1315		6.653
22	5.627	57.98	139.2	0.3586	1.44	56.7	1082
23	5.627	80	206.8		1.308	62.4	1121
24	5.627	35	93.15		1.44	56.7	1078
25	5.627	53	156.2		1.308	62.4	1113
26	1.405	53	156.2		1.308	62.4	1113
27	1.405	35	93.14		1.44	56.7	1078
28	300	133.6	2725		0.1935		134.5
29	300	133.6	561.6		0.1935		22.76

**Table 6:** Overall destroyed exergy rate (total) and destroyed exergy rate for each component in the absorption system.

Equipment	${\dot{B}}_{d}$ (kW)	Relative Values%
Generator	54.4	56.5
Pump	0.001	0.0
Condenser	8.1	8.4
Absorber	23.5	24.4
Evaporator	6.2	6.4
Valves	0.5	0.5
Intermediate heat exchanger	3.6	3.8
Total	96.3	100

## Data Availability

Data is contained within the article or conference paper.

## Abbreviations

The following abbreviations and symbols are used in this manuscript:

COP	air conditioning coefficient of performance
SMER	specific moisture extraction rate (Amount of water removed from the air)
COMPRESSION	Vapor compression cycle
**Symbols:**
B	Exergy of the control volume (J)
$\dot{B}$	Exergy rate (W)
*b*	Specific exergy (kJ/kg)
$\dot{H}$	Enthalpy rate (W)
*h*	Specific enthalpy (kJ/kg)
$\dot{m}$	Mass flow rate (kg/s)
*P*	Pressure (kPa)
$\dot{Q}$	Heat transfer rate (W)
$\dot{S}$	Entropy rate (W/K)
*s*	Specific entropy (kJ/kgK)
*T*	Temperature (${}^{{}^{\circ}}$C)
*t*	Time (s)
*V*	Volume (m${}^{3}$)
*v*	Specific volume (m${}^{3}$/kg)
*x*	mass fraction in the liquid solution (kg${}_{solute}$/kg${}_{solution}$)
$\dot{W}$	Power output (W)
**Greek letters:**
η	efficiency (-)
ρ	specific mass (kg/m${}^{3}$)
**subscripts:**
0	reference state
absorption	absorption refrigeration cycle
compression	vapor compression cycle
CV	control volume
*d*	destroyed
*e*	exit
en	energy basis
evaporator	evaporation unity
desorber	desorber unity
ex	exergy basis
*i*	inlet
